# Radiotherapy in Nigeria: a silent emergency in cancer care

**DOI:** 10.1097/MS9.0000000000004173

**Published:** 2025-10-28

**Authors:** Adeleke Oluwaseun Dorcas, Ikeoluwa Oluwapelumi Ayoola, Divine Besong Arrey Agbor, Toluwalase Awoyemi

**Affiliations:** a Faculty of Medicine, Sumy State University, Sumy, Ukraine; b Bowen University Teaching Hospital, Iwo, Nigeria; c Hematology and Oncology Fellowship, Guthrie Hospital/Robert Parker Hematology and Oncology Fellowship, Sayre, PA, USA; d Division of Cardiology, Department of Medicine, Massachusetts General Hospital, Boston, MA, USA

**Keywords:** Africa, Nigeria, Oncology emergency, Radiotherapy, Radiotherapy equipment

## Abstract

Radiotherapy (RT) remains an essential component of modern cancer management, both for cure and palliation, yet access across Nigeria continues to lag far behind global standards. Despite being Africa’s most populous nation, Nigeria has only a handful of functional RT machines, with frequent breakdowns, erratic power supply, and inadequate maintenance crippling service delivery. Many patients are forced to wait weeks or months for treatment, often with disease progression during the delay. Of the eight government-funded RT centers, only two consistently treat patients with functional linear accelerators. At the same time, most rely on outdated 2D techniques and lack imaging support such as CT or MRI simulators. The shortage of trained personnel exacerbates the crisis: the country lacks clinical residency programs for medical physicists, offers limited opportunities for continuing education, and has an insufficient number of radiation oncologists to meet the growing cancer burden. These limitations not only reduce treatment capacity but also deepen inequities in cancer outcomes, as timely RT becomes a privilege for a few rather than a standard of care. Improving access demands sustained investment, transparent procurement, regular maintenance, and workforce development. International collaboration, public–private partnerships, and strong political will are critical to reversing decades of neglect. The current state of RT in Nigeria is more than a technical shortfall; it is a silent emergency in cancer care that demands immediate attention, strategic planning, and committed implementation.

## Introduction

### Brief overview of the issue

Radiation therapy is used both to cure cancer and to relieve symptoms in advanced cases. It can be combined with surgery, chemotherapy, or immunotherapy, shrinking tumors before surgery (neoadjuvant) or eliminating residual disease afterward (adjuvant). Tumor response varies by type. Radiation is delivered externally (external beam radiation) or internally (brachytherapy). External beams are the most common, using high-energy rays from outside the body. Brachytherapy places radioactive sources directly into the tumor, often used for prostate and gynecological cancers^[1]^.

An estimated 52% of cancer patients will require radiotherapy (RT) at some point during their illness. However, many African countries, including Nigeria, lack adequate RT facilities. Existing centers are often under-resourced in both equipment and personnel. In Nigeria, the majority of cancer cases present at advanced or metastatic stages, frequently requiring palliative RT. Similar patterns are reported across the continent. Given this, the demand for RT in Nigeria is likely to exceed the global estimate. Efforts to improve access began as early as 1968, when Lagos University Teaching Hospital (LUTH) acquired the country’s first superficial RT machine^[2]^.

**HIGHLIGHTS**

- Over half of cancer patients need radiotherapy during their treatment course.
- Nigeria faces severe shortages of radiotherapy machines and trained personnel.
- Equipment breakdowns and power failures cause major treatment delays.
- Most centers rely on outdated 2D techniques with limited modern technology.
- Expanding infrastructure and training is key to improving cancer care access.

According to the DIRAC database, in 2010, there were 13 250 registered RT machines globally, serving a population of approximately 6.68 billion. Access to teletherapy strongly correlated with national income: high-income countries had an average of 8.6 machines per million people, upper-middle-income countries had 1.6, lower-middle-income countries 0.71, and low-income countries just 0.21. These disparities underline the severe shortage of RT in resource-limited settings, particularly in sub-Saharan Africa. Of the 52 African countries analyzed, only 23 had external beam RT facilities, and many of these were under-equipped or poorly staffed. Nigeria, despite being Africa’s most populous nation, had only 7 teletherapy machines at the time – far below the estimated requirement of 145 units to meet the cancer burden. This shortfall contributes to delays in treatment, higher rates of advanced-stage presentation, and poorer outcomes for cancer patients across the region^[3]^.

A 2015 assessment revealed that only two of Nigeria’s nine RT centers operated at full capacity, with just one radiation unit available per 19.4 million people, which is well below global recommendations. Despite the presence of machines, 91.3% of patients experienced treatment delays or cancellations due to systemic issues, such as strikes, power failures, and equipment breakdowns^[4]^. These findings highlight a significant challenge in low- and middle-income countries (LMICs): unreliable access to RT due to poor maintenance and infrastructure, which often prevents patients from completing their treatment and compromises outcomes^[4]^. This editorial is compliant with the TITAN Guidelines 2025^[5]^.

## Current state of RT in Nigeria

Despite the government’s efforts to improve access to RT, it has fallen short on many fronts. A multicenter study of the 8 government-funded RT centers encompassing all 6 geopolitical zones of Nigeria as of 2021 assessed 10 critical areas on the status of RT machines, the RT workforce, and the RT infrastructure in the 8 centers showed that the country has 44 radiation oncologists, 45 residents, 44 medical physicists, and limited other RT personnel, but no medical physics residency programs. Six of the eight RT centers offer radiation oncology residency; only two, National Hospital Abuja (NHA) and Nigeria Sovereign Investment Authority-Lagos University Teaching Hospital (NSIA-LUTH), consistently treat patients with functional linear accelerators (LINACs). Of seven LINACs, only three are functional, and four centers lack any operational teletherapy machines. The Federal Teaching Hospital (FTH) in Gombe does not have either cobalt-60 or LINAC equipment^[6]^.

Nigeria is reportedly equipped with three cobalt-60 machines. There are 9 LINAC machines in West Africa, totaling 43 across the continent^[7]^.

While all centers have brachytherapy machines, only three have functional high-dose-rate (HDR) units. Most centers still use outdated 2D techniques due to a lack of CT/MRI simulators. No center has a PET-CT scanner; only two have a SPECT scanner. Treatment delays are common, and over half of the centers treated few or no patients in 2021. Only FTH, NHA, and NSIA-LUTH reported treatment costs, with NSIA-LUTH (a public–private partnership) charging more than government-subsidized NHA. The absence of pricing standards and inconsistent service delivery further limit access to affordable, effective RT^[6]^.

In improving access to RT, training and retaining the workforce are important steps. There is a notable lack of in-country clinical training programs for medical physicists. In Nigeria, although MSc and PhD programs in medical physics are available, they are largely academic with minimal clinical exposure. While opportunities exist for motivated and promising students to pursue training abroad, funding to support such initiatives remains limited^[8]^.

The lack of training programs in medical physics and funding opportunities to support interested students continues to widen the gap in RT access.

A basic RT center typically requires two teletherapy units, a HDR brachytherapy system, a treatment planning system, a mold room, a simulator, and adequate dosimetry and QA equipment, costing around US$3 million. The IAEA’s Advisory Group on Increasing Access to Radiotherapy Technology initiative promotes affordable RT solutions for LMICs. Recurring costs, including maintenance and source replacement, range from 5.5 to 15% of the initial investment. Cobalt machines are simpler, cheaper to maintain, and use less power, but require source replacement every 5–7 years. Security concerns post-9/11 have increased source replacement costs. In contrast, LINACs have higher maintenance (US$41390 vs. US$5790 for cobalt) and electricity costs^[9]^.

Procurement of RT equipment in Nigeria occurs at both local and national levels, but the process is complex and often lacks transparency. It remains unclear whether the Ministry of Health (MoH) has a structured multi-year procurement plan. Official funding is allocated annually at the national level; however, it is often unreliable and poorly monitored, resulting in a shortfall that fails to meet the needs of RT services. At the local level, some funding is sourced from hospital-generated revenue, but there is significant competition for these limited funds among various departments^[8]^.

The cost of training a team consisting of four radiation oncologists, three medical physicists, and seven radiation therapists is between €1 850 000 and €2 516 000 in Europe^[10]^.

Nigeria has a wait time of 4.0 ± 3.0 weeks for treatment after histological diagnosis to consultation, with an average of 20 newly diagnosed breast, cervical, and prostate cancer cases per month. Nigeria also has a reported wait time of 6.4 ± 5.5 weeks from the Multi-Disciplinary Team discussion to the commencement of actual treatment^[7]^.

A retrospective study done by Adegboyega BC *et al*, in the LUTH, Nigeria on radiation oncology emergencies showed the time from symptom onset to treatment was 24–48 hours in 75 patients (16.4%), between 48 hours and 1 week in 295 patients (64.4%), and more than 1 week in 88 patients (19.2%)^[11]^.

A study by Tumba N, in Northern Nigeria, showed that the overall time from diagnosis to the initiation of RT reflects the total waiting period for treatment. The median waiting time was 30 weeks (IQR 15–51). Patients with breast cancer experienced the longest delays, with a median wait of 47 weeks (IQR 30–66), while those with colorectal cancer had the shortest, with a median of 9 weeks (IQR 5–26)^[12]^.

## Current state

### International community

Nigeria has received significant support from international organizations in developing its RT infrastructure. The International Atomic Energy Agency (IAEA), World Health Organization (WHO), and Japan International Cooperation Agency (JICA) have played vital roles through technical cooperation projects, research programs, fellowships, training courses, seminars, and publications. The IAEA was instrumental in establishing Nigeria’s first three RT centers at LUTH (Lagos), UCH (Ibadan), and ABUTH (Zaria), supplying equipment and training staff. It also facilitated the installation of the country’s first HDR brachytherapy unit. Numerous Nigerian professionals have benefited from IAEA fellowships in radiation oncology, medical physics, radiography, and nuclear medicine. Additionally, JICA has supported manpower development through short-course training in radiation oncology at cancer centers in Japan^[2]^.

## Recommendations/call to action

Addressing the RT crisis in Nigeria requires coordinated short- and long-term strategies involving infrastructure investment, workforce development, and health policy reform. In the short term, efforts should focus on reactivating non-functional machines, standardizing maintenance protocols, and ensuring reliable electricity and power backup systems across centers. A national RT recovery and maintenance task force, led by the MoH, should be established to provide oversight, conduct technical audits, and respond rapidly to equipment failures.

In the long term, infrastructure expansion must be matched with sustainable human resource development. The federal government, in collaboration with teaching hospitals and tertiary institutions, should establish accredited clinical training programs for medical physicists, radiation therapists, and oncology nurses. Scholarships and exchange programs through international partnerships, especially with organizations like the IAEA, WHO, and JICA, should be scaled up to bridge the human resource gap.

Nigeria must also implement a clear national cancer control policy that prioritizes equitable access to RT services across all geopolitical zones. This includes transparent procurement processes, multi-year budget allocations, and public–private partnerships (as seen in NSIA-LUTH) to mobilize resources efficiently. There should be agencies responsible for monitoring the effective implementation of the policy.

The role of non-governmental organizations, philanthropic foundations, and the diaspora medical community is equally vital. These groups can support the establishment of RT hubs, sponsor training, or assist with equipment donations. Additionally, community-based cancer awareness campaigns are necessary to promote early detection, reducing the pressure on late-stage palliative RT services.

Time is of the essence. The government must treat RT infrastructure not as a luxury but as a lifesaving priority. A national RT revitalization agenda, with clear metrics and timelines, is no longer optional; it is essential.

## Conclusion

RT remains one of the cornerstones of effective cancer care, yet in Nigeria, it is chronically neglected. The shortage of machines, trained personnel, and structured policy has turned a treatable diagnosis into a death sentence for many. Despite international support and decades of ineffective interventions, access to RT in Nigeria remains a silent emergency, one that continues to cost lives in delay and despair.

The time for fragmented fixes is over. We need deliberate, urgent, and sustained action. The government must lead, partners must support, and the public must demand better. Cancer does not wait. Neither should we.

## Data Availability

All data gained in the current study are available from the corresponding author on reasonable request.

## Research registration unique identifying number (UIN)

Research registration was not required for this editorial.
